# Gallic acid protects the liver in rats against injuries induced by transient ischemia-reperfusion through regulating microRNAs expressions

**DOI:** 10.22038/ijbms.2018.31589.7605

**Published:** 2019-04

**Authors:** Ghaidafeh Akbari, Feryal Savari, Seyyed Ali Mard, Anahita Rezaie, Mojtaba Moradi

**Affiliations:** 1 Yasuj University of Medical Sciences, Yasuj, Iran; 2 Alimentary Tract Research Center, Physiology Research Center, Department of Physiology, School of Medicine, Ahvaz Jundishapur University of Medical Sciences, Ahvaz, Iran; 3 Department of Pathobiology, School of Veterinary Medicine, Shahid Chamran University of Ahvaz, Ahvaz, Iran

**Keywords:** Gallic acid, Hepatic IR injury, miR-122, miR-34a, Rat

## Abstract

**Objective(s):**

Gallic acid (GA) is a highly effective antioxidant, which its beneficial effects are well known, but its impact on expression of microRNAs (miRs) following hepatic ischemia-reperfusion (I/R) is not well recognized. Therefore, the current research was designed to specify the beneficial effect of GA on miRs (122 and 34a), liver functional tests, and histopathological alterations beyond I/R-induced hepatic injury.

**Materials and Methods:**

Thirty-two rats were randomly divided into four groups (8 per group) including: sham-operated (S), I/R, and GA+I/R pretreated groups. Rats in sham-operated group received physiologic saline (N/S, 2 ml/kg), on a weekly basis, once a day via intraperitoneally route), then a midline abdominal surgery was performed. IR, and GA+IR pretreated groups received physiologic saline (2 ml/kg), and GA (50, and 100 mg per kg) for same time, IP, respectively, before induction of transient ischemia. One hour after reperfusion, biochemical, and histopathological evaluations were performed and expression of miRs were evaluated.

**Results:**

The results showed that GA reduced the concentrations of liver enzymes, miR-122, and miR-34a in serum, and preserved liver cells changes induced by I/R injury.

**Conclusion:**

These findings showed that GA has beneficial effect on liver damage induced by I/R. Therefore, it is suggested that GA can be administered as an anti-miR before elective hepatic surgeries for prevention of this complication.

## Introduction

The clinical conditions caused by hepatic ischemia-reperfusion (I/R) injury are common in liver damage. Ceasing, and reestablishing the hepatic blood circulation during I/R has many deleterious effects on liver function (1). Following hepatic I/R injury, the cell´s scavenging ability decrease, and the accumulation of reactive oxygen species (ROS) activates inflammation, and apoptosis in hepatocytes (2). Therefore, liver protecting against I/R-induced injury by exogenous agents or by increasing the endogenous ability of the liver cells to withstand this insult is important.

Natural agents are preferred to produce new effective medicinal product (3). It is well-documented that ﬂavonoids, anthocyanin and other phenolic compounds are responsible for the beneficial effects of plants (4). These agents have many pharmacological benefits. In this regard, gallic acid (GA) received much attention due to its strong antioxidant property (5).

**Figure 1:**
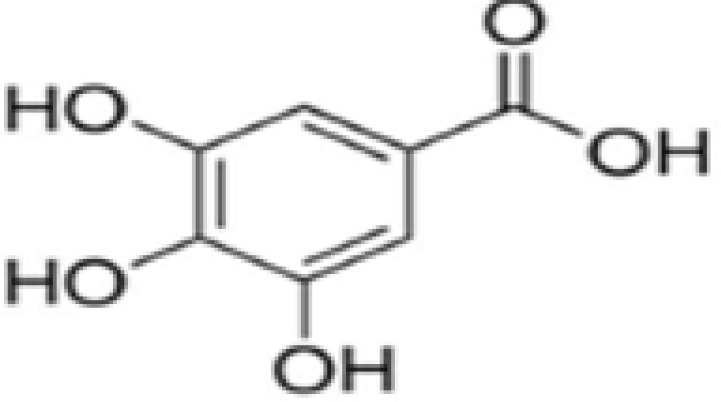
Chemical structure of gallic acid (GA; 3,4,5-trihydroxybenzoicacid)

GA or 3,4,5-trihydroxybenzoic acid (Figure 1) is considered as a potent, and well-known antioxidant (6). This antioxidant with yellowish white crystal has a molecular mass of 170.12 g/mol, and melting point of 250 ^°^C. It dissolves in water at 20 ^°^C (7). The biological effects of this antioxidant include antioxidant (8), anti-allergic- (9), anti-microbial (10), anti-cancer (11), anti-ulcer (12), and neuroprotective effects (13).

The results of a study showed that GA through increasing the potency of endogenous antioxidant system protects the isolated heart of rat against I/R induced injury (14). Moreover, the tissue protective ability of GA against injuries induced by I/R is well-proven (15). The active radicals of oxygen are a category of oxidants, which are produced endogenously as a byproduct from enzymatic reactions in mammalian body (16). In addition, recent reports have shown that ROS are able to upregulate microRNAs (miRs), which in turn regulate the transcription factor (17, 18). MiR-122 is known as the most abundant, specific and important miR in the liver. It plays many physiologic roles in this organ (19, 20). Another related and important miR that represents the liver injury is miR-34 (21, 22). 

As mentioned earlier, the liver protective activity of GA on paracetamol (23), CCL4 (24), and I/R-induced injuries has been reported (25), but its impact on serum concentrations of miR-122, and miR-34a beyond I/R-induced hepatic injury has not been determined. Therefore, the goals of this research study were 1. To determine the hepatoprotective effect of GA pretreatment on I/R- induced hepatic injury; and 2. To test its effect on expression of the above-mentioned miRs after I/R-induced liver injury. 

## Materials and Methods

***Animals***

In this animal study, male, Wistar rats weighing 200–250 g were used. Animals were purchased from the animal center of Ahvaz Jundishapur University of Medical Sciences, Ahvaz, Iran. Rats were nourished with a standard diets and tap water *ad libitum* and were kept under standard situations of humidity, temperature (20-24 ^°^C), and 12-hr light–dark cycle. Animals were fasted overnight before performing the I/R procedure. All procedures including anesthesia, surgery, and blood withdrawing were performed in accordance with ethics committee of Ahvaz Jundishapur University of Medical Sciences (IR.AJUMS.REC.1396.281).

***Animal grouping***

In this experiment, 32 rats were divided into four experimental groups (8 in each). 1.Sham-operated: Animals in this group received an intraperitoneal injection of physiologic saline (N/S; 2 ml per day) on a daily basis for a week (26), then laparotomy was performed; 2. I/R group: animals in this group received the same dose of physiologic saline via the same route, then on the day 8, I/R induction was performed**,** and groups 3 and 4.GA pretreated+ I/R groups (GA+I/R): Rats received GA (50, or 100 mg/kg, IP) (25) on a daily basis for a week, then on the day 8, I/R induction was carried out. 

**Figure 2:**
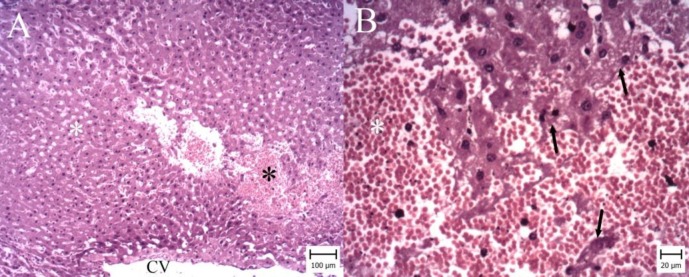
Liver. Ischemia-reperfusion (I/R) group. (Hematoxylin and Eosin). A: Note to large area of necrosis (White asterisk) and hemorrhage (Black asterisk) around central vein (CV) (Bar:100 µm). B: Necrotic cells are observed with dark nuclei and cytoplasm (arrows) and many erythrocytes filled the spaces between necrotic hepatocytes (asterisk)(Bar:20 µm)

**Figure 3:**
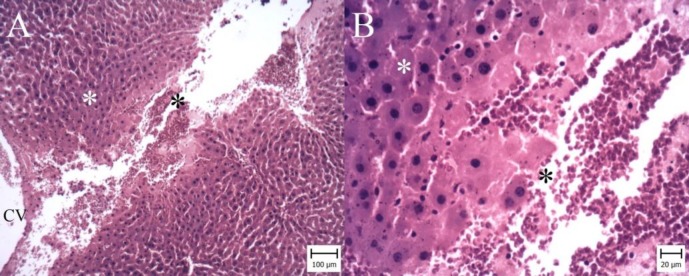
Liver. Gallic acid 50 mg per kg (GA50) group. (Hematoxylin and Eosin). A: Necrotic area (White asterisk) and hemorrhage are observed around central vein (CV) (Bar: 100 µm). B: Note to necrotic hepatocytes (White asterisk) and erythrocytes (Black asterisk) (Bar: 20 µm)

**Figure 4:**
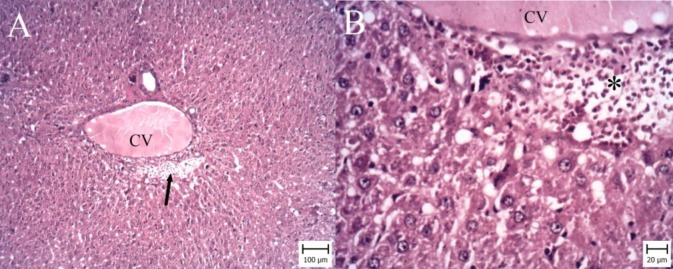
Liver. Gallic acid 100 mg per kg (GA100) group. (Hematoxylin and Eosin). A: Note to small area of hemorrhage around central vein (CV) (arrow) (Bar: 100 µm). B: Erythrocytes (asterisk) are accumulated around CV (Bar: 20 µm)

**Figure 5:**
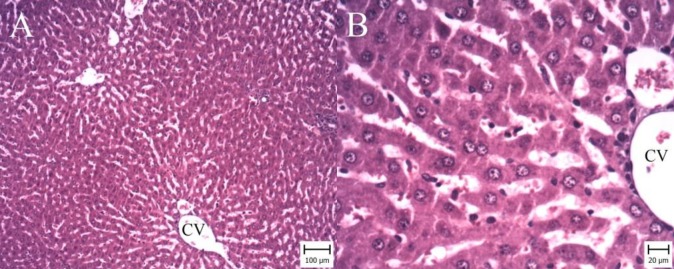
Liver. Sham group. (Hematoxylin and Eosin). A: Note to normal structure of hepatocytes around central vein (CV) (Bar: 100 µm). B: Hepatocytes with normal nuclei and cytoplasm are situated around CV (Bar: 20 µm)

**Figure 6:**
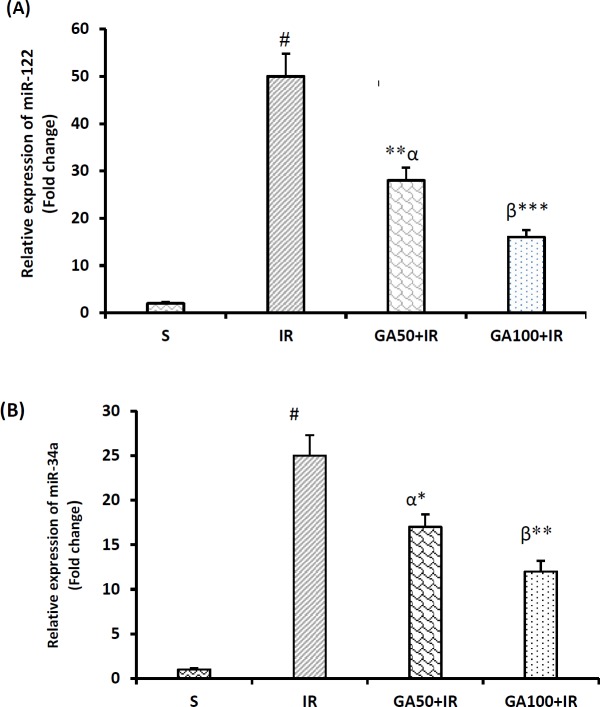
Gallic acid improves the expression levels of miR-122(A) and miR-34a (B), after hepatic I/R injury. β*P*<0.01, α*P*<0.001 and #*P*<0.0001significant difference compared to the sham-operated group. *P<0.05,**P<0.01and ***P<0.001 significant difference compared to the I/R group. I/R: Ischemia/reperfusion; S: Sham; GA: Gallic acid; GA+IR: Gallic acid pretreated I/R groups

**Figure 7:**
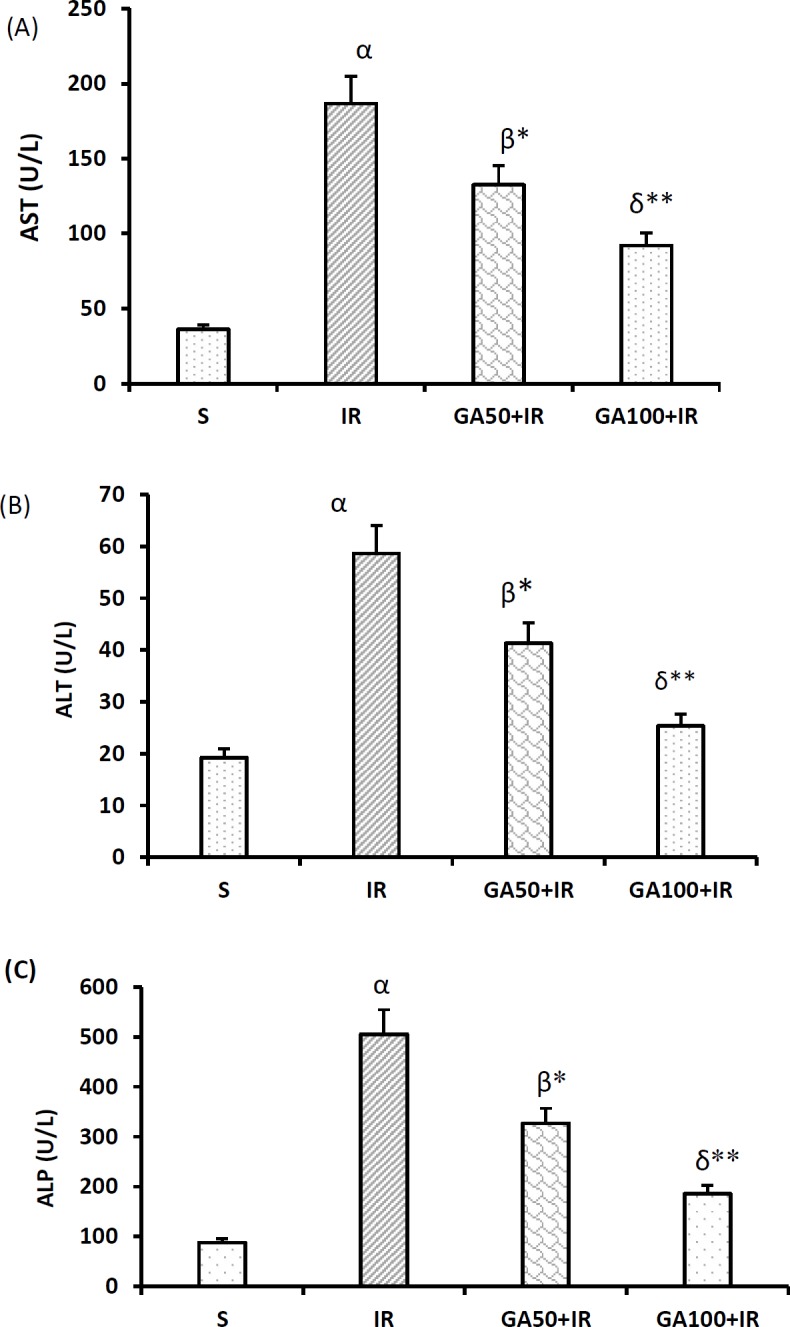
Gallic acid lowered the sera levels of hepatic transaminases, and ALP after hepatic I/R injury. Data represented as mean±SEM, 8 rats in each group. αP<0.001, β*P*<0.01 and δ*P*<0.05 significant difference compared to the sham-operated group. *P<0.05, **P<0.01 and ***P<0.001 significant difference compared to the I/R group. I/R: Ischemia/reperfusion;AST: Aspartate aminotransferase; ALT: Alanine aminotransferase; ALP: Alkaline phosphatase; U/l: Unit per liter

**Figure 8:**
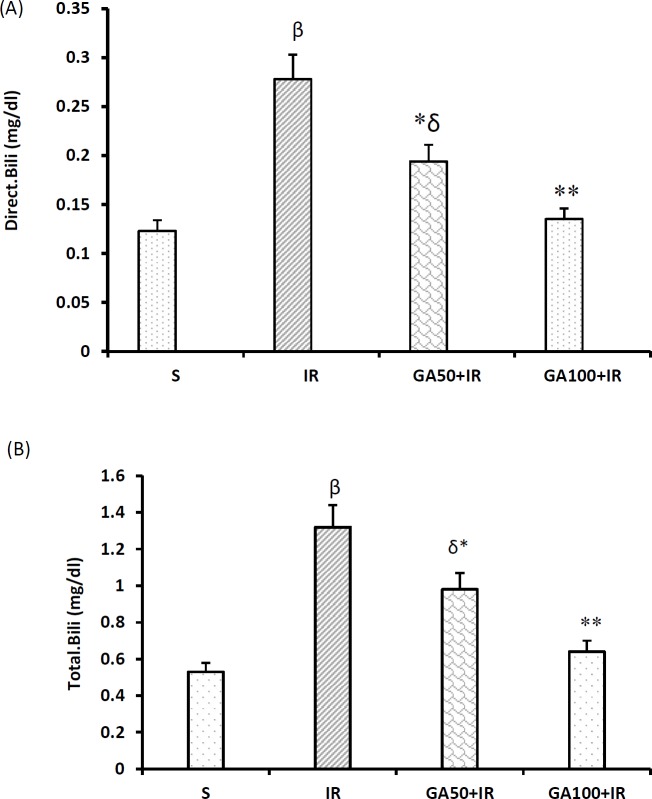
Gallic acid improved the serum concentrations of direct and total bilirubin following hepatic I/R injury. Results represented as mean±SEM, 8 rats in each group. β*P*<0.01 and δ*P*<0.05 significant difference compared to the sham group. *P<0.05 and **P<0.01 significant difference compared to the I/R group. I/R: Ischemia/reperfusion; mg/dl: miligram/deciliter

***Surgical procedure***

Animals were anesthetized by IP injection of ketamine at 80 mg per kg and xylazine at 10 mg per kg (Alfasan Co. Woerden-Holland). Duration of ischemia episode was 45 min and after that the arterial clamp was removed to establish reperfusion for 60 min (27). After finishing the reperfusion episode, animal were euthanized by cardiac exsanguination and small liver parts were fixed with 10% formalin.

***Molecular evaluation***

To evaluate the expression of miRs, first the frozen serum samples were melted in room temperature and then extraction procedure was performed using manufacturer’s protocol [miRNeasy/Plasma kit; QIAGEN, GmbH, Germany]. After that, the concentration and purity of extracted RNA was checked using a nanodrop (Nanodrop thermo scientific S.N:D015). At the next step, 1 microgram of extracted RNA was used for synthesis of complementary DNA (cDNA) by using miScript II RT kit (QIAGEN, GmbH, Germany). 

***MicroRNAs amplification***

miRs were amplified by quantitative real-time polymerase chain reaction (qRT-PCR) using a Light Cycler® 96 Real time PCR System (Roche Diagnostics, Indianapolis, IN, USA). The final volume for each PCR reaction was 20 µl containing 2 µl cDNA, 10 µl 2× QuantiTect SYBR Green PCR Master Mix, 2 µl 10× miScript Primer Assay [miR-122 (MS00000315), or miR-34a (MS00000224); QIAGEN], 2 µl 10×miScript Universal Primer [(MS0003374); (QIAGEN)], and 4 µl RNAase free water. The time, and temperature table for PCR reaction was as follow: 15 min at 95 ^°^C to activate HotStar Taq DNA polymerase as initial step and then followed by 45 cycles at 94 ^°^C for 15 sec, 55 ^°^C for 30 sec, and 70 ^°^C for 30 sec. In addition, a no-template negative control (H_2_O) was routinely run in every PCR reaction. The changes in miRs expression levels in serum were normalized using housekeeping miRs, RNU6 and the fold change was determined using the 2^–ΔΔCt^ formula. 

***Biochemical assay of hepatic transaminases***

After centrifuging (3000 rpm for 10 minutes) of blood samples, serum was separated, and kept at -20 ^°^C until analysis. The serum concentrations of hepatic transaminases were measured with commercial kits (Pars Azmoon; IR, Iran) according to the manufacturer’s instructions using a serum autoanalyzer (BT-1500-A-A, Rome Italy).

***Histopathological analysis***

Formalin-fixed liver tissues were processed as routine in histopathology lab. When the slides stained by Haematoxylin and Eosin, they were assessed by light microscope. 

***Data analysis***

Statistical tests, one-way analysis of variance (ANOVA) and Dunnett’s or LSD *post hoc* tests were used for analyzing the data. Results are shown as mean±standard errors of the means (SEMs). *P*<0.05 was considered statistically significant.

## Results

***Gallic acid improved histopathological changes***

According to microscopic inspection, large area of necrosis and hemorrhage were observed in the liver of I/R group (Figure 2A). Necrotic hepatocytes with dark nuclei and cytoplasm were observed and erythrocytes were filled the spaces between necrotic hepatocytes (Figure 2B). In GA50 group, the area of necrosis and hemorrhage were smaller (Figure 3). In GA100 group, the area was very small and the size of necrosis and hemorrhage reduced (Figure 4). GA100 mg/kg had better effect than GA50 mg/kg on these parameters. Structure of liver in sham group was normal (Figure 5).

***Gallic acid improved the serum concentrations of the studied microRNAs***

Figures 6A, and 6B show that miR-122 and miR-34a in serum were elevated after induction of hepatic I/R injury as compared to sham-operated rats (*P*<0.0001 in both cases). The expression levels of these miRs in all experimental groups were lower than I/R group (*P*<0.05). Both studied doses of GA [50, and 100 mg/kg] significantly decreased these levels (*P*<0.05, and *P*<0.01, respectively). However, GA at higher dose [100 mg/kg] had better effect than GA50 mg/kg on the levels of these miRs (*P*<0.001, and *P*<0.01, respectively) in relative to I/R group. 

***Effect of gallic acid pretreatment on hepatic transaminases, and alkaline phosphatase level***

Figures 7A, B, and C show that the sera concentrations of hepatic transaminases, and alkaline phosphatase (ALP) increased following liver I/R injury. These levels in I/R group were higher than sham group (*P*<0.001). GA decreased these enzymes in all studied groups (*P*<0.05). Also, GA at higher dose (100 mg/kg) had better effect than low dose (50 mg/kg) (all cases were *P*<0.001). 

***Gallic acid controlled the adverse effect of liver I/R injury on sera concentrations of direct, and total bilirubin***

Induction of liver I/R injury increased the serum concentrations of direct, and total bilirubin after I/R injury (Figures 8A, and 8B) (*P*<0.001). Figures 8A, and 8B also show that both studied doses of GA prevented these increments (*P*<0.05). However, the higher studied dose of GA had more efficacy (both *P*<0.01).

## Discussion

This research study showed GA was able to mitigate deleterious effects of transient hepatic ischemia in rats by a) decrementing the expression of miRs [122, and 34a]; and b) attenuating the levels of hepatic transaminases, ALP, and total, and direct bilirubin in serum. 

Our qRT-PCR results were in consistent with previous studies (28, 29) indicating that the expression levels of miRs [122, and 34a] enhanced after inducing liver I/R injury. Both studied doses of GA prevented the increment of miR-122. Recent reports have been pointed out that determination of the serum level of miR-122 is more important than measuring the levels of liver functional enzymes, because its plasma level increases more rapid than the plasma concentrations of hepatic transaminases, in response to liver injury (21, 30). Therefore, in terms of clinical significance, measuring the plasma level of this biomarker provide a fast, easy, reliable and non-invasive way for diagnosis and prognosis of liver disorder (31). Therefore, the inhibitory effect of GA on miR-122 expression along with its liver protective effect as shown by the present histopathological findings showed that the first effect was secondary to their cytoprotective effects. 

Consistent with previous research (29, 32), the present findings demonstrated that inducing ischemia and then reperfusion increases the serum concentration of miR-34a. This increase could be a result of the overproduction of ROS beyond hepatic I/R insult (17, 18, 33, 34). In agreement, a study reported that the increase of the expression of miR-34a was in parallel with age-related loss of liver antioxidant system (35). A recent study showed that the expression of miR-34a increases in nonalcoholic steatohepatitis [NASH] patients, and confirmed the validity of miR-34a in diagnosing NASH in comparison with alanine aminotransferase (ALT) in patient with nonalcoholic fatty liver disease (NAFLD) (36). Therefore, they concluded that measuring plasma level of miR-34 could be a disease-specific noninvasive biomarker for the diagnosis of NASH. Our results revealed that pretreatment with GA for seven days significantly prevented the hepatic I/R-induced miR-34 expression. In our previous report, it has been shown that crocin as a strong antioxidant, through silencing the gene expression of miR-34, had a significant hepatoprotective activity against I/R injury in rat (29).

All previous literatures on the effect of GA on liver have shown that GA protected the liver tissue against I/R-, paracetamol-, and CCl4-induced injuries through exerting significant antioxidant, and ROS scavenging properties (24, 25). These findings together concluded that antioxidants such as GA, and carnosic acid (37), through scavenging ROS, inhibit ROS effects on miR-34a expression and their final effects would be silencing the gene expression of miR-34a and protecting the liver of rats.

It has been shown that membrane-stability of the liver cells is affected by I/R injury. The present microscopic findings showed that following I/R injury, cell membranes lost their integrity. Therefore, the cause of the increment of aspartate aminotransferase (AST), ALT, and ALP in plasma could be losing the cell membranes integrity. It has been shown that GA, through stabilizing the cell membrane due to its antioxidant property, protected the liver against paracetamol (23). 

The sera concentrations of liver transaminases, and ALP have been reported to enhance after I/R injury (38). This increase may in part be due to losing the hepatocyte membranes integrity as confirmed by the histopathological findings. Administration of GA at doses 50 mg/kg, and 100 mg/kg prevented the increment of plasma levels of transaminases, and ALP.

The reversal influence of GA on I/R-induced increment of liver enzymes in serum may be resulted from the membrane-stabilizing and antioxidant activity (23), which was supported by the restricted extent of histological changes.

This research achieved better results than Bayramouglu *et al.* study, in which the plasma concentrations of liver transaminases and ALP decreased about 50% in animals receiving GA at dose 100 mg/kg compared to I/R group, while our results showed more inhibitory effect of GA at dose 100 mg/kg on these parameters. It seems that time course of administration had a major role in achieving these results. We pretreated GA for 7 consecutive days before I/R induction, while in the study of Bayramouglu *et al. *it was administered once. Therefore, time course of administration was a key factor to achieve the desirable results. Moreover, it has been shown that these effects of GA following I/R injury may be resulted from the protection of sinusoidal endothelial cells, which are the first target of improvement of sinusoidal blood supply by GA (25).

Therefore, inhibition or modulating the expression of miRs by GA, as shown in the current study or other compound such as hydrogen sulfide as reported in a previous study (39), can effectively treat liver diseases.

## Conclusion

This study showed that GA had potential hepatoprotective role on I/R-induced injury. Therefore, these data suggest that GA can be administered as an anti-miR before elective hepatic surgeries for prevention of this complication. 
